# Colorectal cancer health services research study protocol: the CCR-CARESS observational prospective cohort project

**DOI:** 10.1186/s12885-016-2475-y

**Published:** 2016-07-08

**Authors:** José M. Quintana, Nerea Gonzalez, Ane Anton-Ladislao, Maximino Redondo, Marisa Bare, Nerea Fernandez de Larrea, Eduardo Briones, Antonio Escobar, Cristina Sarasqueta, Susana Garcia-Gutierrez, Urko Aguirre, for the REDISSEC-CARESS/CCR group, Jose María Quintana, Marisa Baré, Maximino Redondo, Eduardo Briones, Nerea Fernández de Larrea, Cristina Sarasqueta, Antonio Escobar, Francisco Rivas, Maria M. Morales-Suárez-, Juan Antonio Blasco, Isabel del Cura, Inmaculada Arostegui, Amaia Bilbao, Nerea González, Susana García-Gutiérrez, Iratxe Lafuente, Urko Aguirre, Miren Orive, Josune Martin, Ane Antón-Ladislao, Núria Torà, Marina Pont, María Purificación Martínez del Prado, Alberto Loizate, Ignacio Zabalza, José Errasti, Antonio Z Gimeno, Santiago Lázaro, Mercè Comas, Jose María Enríquez, Carlos Placer, Amaia Perales, Iñaki Urkidi, Jose María Erro, Enrique Cormenzana, Adelaida Lacasta, Pep Piera Pibernat, Elena Campano, Ana Isabel Sotelo, Segundo Gómez-Abril, F. Medina-Cano, Julia Alcaide, Arturo Del Rey-Moreno, Manuel Jesús Alcántara, Rafael Campo, Alex Casalots, Carles Pericay, Maria José Gil, Miquel Pera, Pablo Collera, Josep Alfons Espinàs, Mercedes Martínez, Mireia Espallargues, Caridad Almazán, Paula Dujovne Lindenbaum, José María Fernández-Cebrián, Rocío Anula Fernández, Julio Ángel Mayol, Ramón Cantero, Héctor Guadalajara, María Heras, Damián García, Mariel Morey, Javier Mar

**Affiliations:** Unidad de Investigación, Hospital Galdakao-Usansolo, Galdakao, Bizkaia Spain; Unidad de Investigación, Hospital Costa del Sol, Málaga, Spain; Unidad de Epidemiología Clínica, Corporacio Parc Tauli, Barcelona, Spain; Departamento de Salud, Madrid, Spain; UDG Salud Publica, Distrito AP Sevilla, Sevilla, Spain; Unidad de Investigación, Hospital Basurto, Bilbao, Bizkaia Spain; Unidad de Investigación, Hospital Donosti, Donostia-San Sebastian, Gipuzkoa, Spain; Red de Investigación en Servicios de Salud en Enfermedades Crónicas (REDISSEC), Galdakao, Bizkaia Spain

**Keywords:** Colon cancer, Rectal cancer, Clinical prediction rule, Health services research

## Abstract

**Background:**

Colorectal cancers are one of the most common forms of malignancy worldwide. But two significant areas of research less studied deserve attention: health services use and development of patient stratification risk tools for these patients.

**Methods:**

Design: a prospective multicenter cohort study with a follow up period of up to 5 years after surgical intervention. Participant centers: 22 hospitals representing six autonomous communities of Spain. Participants/Study population: Patients diagnosed with colorectal cancer that have undergone surgical intervention and have consented to participate in the study between June 2010 and December 2012. Variables collected include pre-intervention background, sociodemographic parameters, hospital admission records, biological and clinical parameters, treatment information, and outcomes up to 5 years after surgical intervention. Patients completed the following questionnaires prior to surgery and in the follow up period: EuroQol-5D, EORTC QLQ-C30 (The European Organization for Research and Treatment of Cancer quality of life questionnaire) and QLQ-CR29 (module for colorectal cancer), the Duke Functional Social Support Questionnaire, the Hospital Anxiety and Depression Scale, and the Barthel Index. The main endpoints of the study are mortality, tumor recurrence, major complications, readmissions, and changes in health-related quality of life at 30 days and at 1, 2, 3 and 5 years after surgical intervention.

Statistical analysis: In relation to the different endpoints, predictive models will be used by means of multivariate logistic models, Cox or linear mixed-effects regression models. Simulation models for the prediction of discrete events in the long term will also be used, and an economic evaluation of different treatment strategies will be performed through the use of generalized linear models.

**Discussion:**

The identification of potential risk factors for adverse events may help clinicians in the clinical decision making process. Also, the follow up by 5 years of this large cohort of patients may provide useful information to answer different health services research questions.

**Trial registration:**

ClinicalTrials.gov Identifier: NCT02488161. Registration date: June 16, 2015.

**Electronic supplementary material:**

The online version of this article (doi:10.1186/s12885-016-2475-y) contains supplementary material, which is available to authorized users.

## Background

Colorectal cancers are currently among the most common cancers in both women and men [1, 2]. Although many scientific approaches have been applied in this field, primarily with respect to diagnosis and treatment, two significant aspects require increased focus, the first of which is the development of clinical prediction rules to predict adverse events after surgical treatment of colorectal cancer patients. While some measurements of short-term outcomes have been developed, such as the different versions of the Physiological and Operative Severity Score for the enUmeration of Mortality and morbidity (POSSUM) scoring [3–5], most are not properly validated in other settings. Furthermore, prediction models for medium-term follow up (e.g., 1 or 2 years), a period in which the majority of adverse outcomes after treatments are observed, are inadequate. Not only robust clinical outcomes such as mortality, major complications, relapses or reinterventions should be studied and related factors identified, but also the determinants of changes in health-related quality of life, as perceived by the patient; these are referred to as patient reported outcome measurements (PROMs) [6–8]. The second significant area of research interest is related to health services research, where multiple factors remain unquantified. Among these are the quality of the health care process from the time that initial symptoms present in the patient to the time at which the patient receives treatment, equity in access to diagnosis and treatment procedures, provision of auxiliary support services (including key psychosocial services) or the cost and effectiveness of different surgical approaches [9–11].

Study protocol manuscripts are quite common in the cancer research field but mainly to present clinical trials of treatments for these patients. Less common, though no rare, other large and ambitious studies dealing with quality of care o even health services research have also published their study protocol. [12, 13].

### Study goals and objectives

**Fig. 1:**
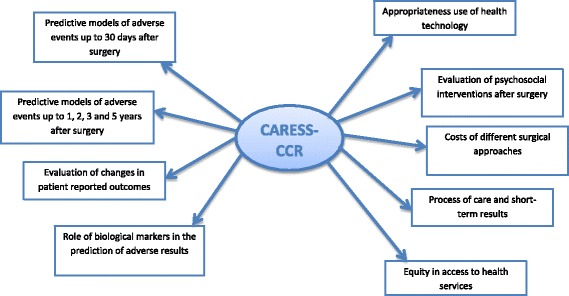
Study objectives

The purpose of this study is to clarify some of the previously stated questions and unknown factors. The specific study objectives are displayed in Fig. 1 and are: 1. To determine risk factors for death or major complications, reoperations, or readmissions in the short-term; 2. To determine risk factors for death, tumor recurrence, major complications, readmission or deterioration in quality of life in the mid-term; 3. to evaluate patient reported outcomes from before intervention to the end of the follow-up period. 4. To evaluate the role of biological markers in the prediction of adverse results; and 5. from a health services research perspective, to evaluate equity, appropriateness, access to care, treatment cost, and psychosocial support for patients.

With the purpose of reducing publication bias and improving reproducibility and to serve as reference for future manuscripts derived from this study and for readers we present the study protocol of this multicenter research work.

## Methods/design

### Study design

An observational analytic prospective cohort study with a planned patient follow up period of 5 years following surgical intervention.

Setting: patients recruited from 22 hospitals representing 9 provinces in Spain, all of which operate under the Spanish National Health Service (SNHS), which is responsible for the majority of the national population. All applicable residents have free access to their primary care physician and to the hospital ED. Participating hospitals are: Hospital de Antequera, Hospital Costa del Sol, Hospital Universitario de Valme, Hospital Universitario Virgen del Rocío, Hospital Universitario Virgen de las Nieves, Hospital Universitario de Canarias, Corporació Sanitaria Parc Taulí, Althaia, Hospital del Mar, Hospital Clínico San Carlos, Hospital Universitario La Paz, Hospital Infanta Sofía, Hospital Universitario Fundación Alcorcón, Hospital Galdakao-Usansolo, Hospital Universitario Araba, Hospital Universitario Basurto, Hospital Universitario Cruces, Hospital Hospitalario Donostia, Hospital Bidasoa, Hospital de Mendaro, Hospital de Zumarraga and Hospital Universitario Doctor Peset.

Research population: Patients diagnosed with colorectal cancer that underwent surgical intervention at one of the listed hospitals between June 2010 and December 2012. Patients were informed of the study objectives and invited to voluntarily participate. Patients were included in the study sequentially.

Expected duration of the study: patients will be followed until 5 years after the surgical index intervention, or until death.

#### Selection criteria

Patients will be deemed eligible for the study if they have undergone surgery for colorectal cancer in one of the participating hospitals. Colorectal cancer diagnosis is based on anatomopathological diagnosis after a biopsy by colonoscopy [10, 14, 15].

**Fig. 2:**
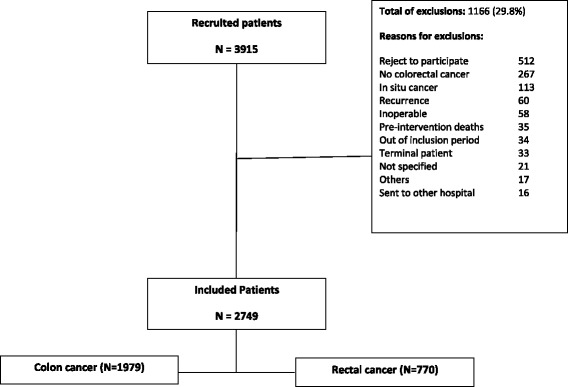
Flow chart of the recruitment process

Inclusion criteria: diagnosis with colon cancer (up to 15 cm above the anal margin) and rectum (between the anal margin and 15 cm above it), where curative or palliative surgery by first time was applied. Exclusion criteria are colon or rectum in situ cancer, inoperable tumor, severe mental or physical condition which prevents the patient from responding to questionnaires, terminal illness, inability to respond to questionnaires for any reason, or lack of consent to participate in the study. Figure 2 illustrates a flow chart of the recruitment process. Recruitment data from participating hospitals as of December 2012 are provided in Table 1.

**Table 1:** Recruited patients by area and hospital

Autonomous community	Hospital	Valid patients
		*n*
Andalucía	Antequera	41
	Costa del Sol	95
*n* = 490	Valme	137
	Virgen de las Nieves	31
	Virgen del Rocío	186
Canarias *n* = 101	Complejo Hospitalario de Canarias	101
Cataluña	Corporació Parc Taulí	332
*n* = 689	Althaia	100
	Hospital del Mar	257
Madrid	La Paz	169
	Infanta Sofía	64
*n* = 343		
	Clínico de San Carlos	39
	Alcorcón	71
País Vasco	Txagorritxu	88
	Bidasoa	33
	Donostia	245
	Mendaro	28
*n* = 998	Zumarraga	39
	Basurto	229
	Cruces	139
	Galdakao-Usansolo	197
Valencia *n* = 128	Doctor Pesset	128
Total		2749

### Methodology

Eligible patients were primarily identified from the surgical waiting lists of participating hospitals. For those receiving urgent surgery, identification took place during the hospitalization period. Depending on the hospital, one of two strategies were implemented for the inclusion of patients: a) surgeons or other healthcare professionals explained the study objectives and invited patients to voluntarily participate, during a clinical visit prior to surgery or during hospitalization for surgery; or b) written information about the goals of the study and the consent form for participation were sent to patients prior to hospitalization for surgery.

**Fig. 3:**
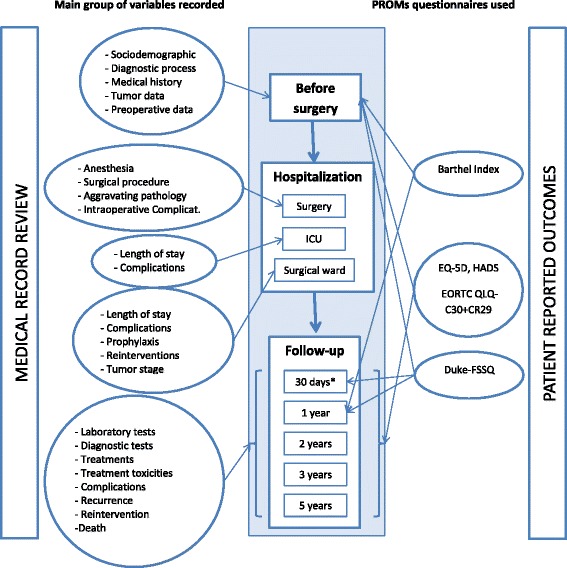
Data gathering process from baseline to 5 years of follow up. * Patient reported outcomes measures (PROMs) used: EuroQol-5D (EQ-5D), EORTC QLQ-C30 (European Organization for Research and Treatment of Cancer Quality of Life Questionnaire-C30); QLQ-CR29 (European Organization for Research and Treatment of Cancer Questionnaire Module for Colorectal Cancer), the Duke-UNC Functional Social Support Questionnaire (FSSQ), the Hospital Anxiety and Depression Scale (HADS), and the activities of daily living (ADL) Barthel Index (BI). Patient reported outcome at 30 days were collected in a sub-sample of the overall cohort and after 2 years in a selection of the original hospitals. ICU: Intensive Care Unit

Following the patient selection process, clinical data and PROMs were collected at specific time points. Figure 3 summarizes the data-gathering process.

### Data collection

A. Medical records and direct data collection

Clinical data were gathered from medical records and databases by qualified reviewers. A data collection form and instruction manual were developed to guide the data collection process to ensure consistency among centers and reviewers.

Upon hospital admission, baseline data were collected that included information on patient sociodemographics, clinical data (including data from the initial diagnosis, the pre-intervention process, onset of symptoms, habits and lifestyles (physical activities, alcohol intake or smoking habit), personal and family background, general comorbidity data as recorded in the Charlson Comorbidity Index [16], bowel-specific comorbidities, diagnostic and screening tests, pre-intervention treatments, and duration between relevant time points in the diagnostic process), preoperative data [including analytical, tumor markers, diagnostic tests and pre-intervention clinical staging (pre-TNM), and tumor site], data from the outpatient anesthesia visit prior to surgical intervention (ASA stage [17], physiological and cardio-respiratory parameters and tests, and consciousness level based on the Glasgow scale [18]). Variables from the different versions of the POSSUM were specifically included to test all those rules.

### Data related to hospital admission

- Data on the surgical intervention (including type of intervention, surgical severity, aggravate diagnosis, type of anesthesia, antibiotic prophylaxis, surgical approach, type of surgery, invasion of adjacent organs, additional surgical procedures associated to local extension of the disease, intra surgical complications, complication grade, duration of surgery, and other information specific to the colon- or recto-surgical intervention), and Intensive Care Unit (ICU) period information (duration, mechanical ventilation requirements, parenteral nutrition, antibiotics, presence of complications, and death).
- Anatomical pathology data, including tumor-free margin, histologic diagnosis, pre-TNM (pTNM), lymph nodes analyzed and affected, degree of tumor differentiation, invasion, remission, and biological markers.

In relation to the biological marker objective of the study, 800 paraffin-embedded samples were collected for immunohistochemical analysis to identify clinical and pathological features associated with changes proliferation, apoptosis and angiogenesis, and the role of these changes in the occurrence of adverse results such as cancer relapse and mortality. Samples were collected from patients attending the following hospitals: Hospital de Antequera, Hospital Costa del Sol, Hospital de Valme, Hospital Virgen del Rocío, Hospital Virgen de las Nieves, Hospital Galdakao-Usansolo, and Hospital Basurto.

c. Other data related to hospital admission (length of stay, presence and degree of complications, treatment information, need for reintervention, or death). Complications were grouped in the following categories: surgical, medical, infectious, and hematological (hemorrhage/thrombosis/embolism) (see Additional file 1: Table S1).

Data were subsequently collected up to 30 days after surgery (analytics, diagnostic tests, presence of complications, readmissions, reintervention, social service requirements, or death). Oncologic treatment information is shown in the adittional files section as Table 2.

**Table 2:** Basic description of the whole sample at baseline

	*N* (%)	Main cancer location	
		Rectal	Colon
Total	2749	770 (28.01)	1979 (71.99)
Gender (Men)	1749 (63.62)	522 (67.79)	1227 (62.00)
Age^a^	68.50 (10.97)	67.14 (11.26)	69.02 (10.81)
Charlson Comorbidity index^a^	2.88 (1.29)	2.83 (1.22)	2.90 (1.32)
ASA index			
I,II	1548 (57.92)	450 (59.92)	1098 (57.13)
III	1020 (38.16)	277 (36.88)	743 (38.66)
IV	105 (3.93)	24 (3.20)	81 (4.21)
Surgical approach (main)			
Open surgery	1139 (41.89)	307 (40.55)	832 (42.41)
Laparoscopy	1580 (58.11)	450 (59.45)	1130 (57.59)
Laterality of the tumor (Right –transverse side)	834 (42.14)	NA	834 (42.14)
pTNM			
0, I, II	1581 (57.85)	485 (63.32)	1096 (55.72)
III	892 (32.64)	221 (28.85)	671 (34.11)
IV	260 (9.51)	60 (7.83)	200 (10.17)

Laterality of the tumor: Right-transverse side (appendix, cecum, ascending colon, right hepatic flexure and transverse) versus left side (left splenic flexure, descending colon, and sigmoid colon)

*ASA*, American Society of Anesthesiologists

*pTNM* pathological tumor-node-metastasis staging

^a^Means and, in brackets, standard deviation

Information was next collected through the first year of follow-up, including need for and type of treatment (separately evaluated for colon or rectal tumors), radiation therapy, chemotherapy (treatment schedule, neo or adjuvant therapy, number of cycles, presence and degree of treatment complications, and supportive care requirements), other medical treatments and psychological support. Information was also collected on laboratory and diagnostic tests performed, presence of complications, additional surgeries, tumor recurrence, readmission or reintervention, and death.

Information collected at the 2-, 3- and 5-year visits was similar to that of the 1-year visit.

B. Patient reported outcomes measures (PROMs)

Patients completed the following questionnaires prior to surgery and in the follow up after surgery: EuroQol-5D (EQ-5D), EORTC QLQ-C30 (The European Organization for Research and Treatment of Cancer Quality of Life Questionnaire-C30) and QLQ-CR29 (European Organization for Research and Treatment of Cancer Questionnaire Module for Colorectal Cancer), the Duke-UNC Functional Social Support Questionnaire (FSSQ), the Hospital Anxiety and Depression Scale (HADS), and the activities of daily living (ADL) Barthel Index (BI).

The EQ-5D is a generic health-related quality of life (HRQoL) questionnaire which consists of two parts, the first of which is a description of the state of health in five dimensions: mobility, self-care, usual activities, pain/discomfort and anxiety/depression. Each of these dimensions is measured by three answer options that define different levels of severity. The second part is a visual analogue scale in which patients rate their health on a scale displayed in the form of a thermometer (20 mm), whose ends are 0 (worst imaginable health state) to 100 (best state imaginable health) [19]. This questionnaire has been translated into and validated in Spanish [20].

The EORTC QLQ-C30 is a HRQoL questionnaire for evaluating the for cancer patients undergoing treatment, and has shown good validity and reliability in its Spanish adaptation (Cronbach’s alpha coefficient of 0.7). It consists of 30 items, 28 of which have four possible answers (not at all, a little, quite a bit, very much) and two items with seven response options (a visual analogue scale where one is “very poor” and seven “excellent”) [21, 22]. The timeframe assessed refers to the previous week. The scores on each scale are transformed so that the final values are between 0 and 100. In addition to this overall score, the instrument consists of three scales: Global health status, in which a high score represents a high performance in terms of overall health; Functional–scale, which consists of five subscales of operation: physical, role, emotional, cognitive and social; and a Symptoms scale, describing a number of symptoms: fatigue, nausea, vomiting, pain, dyspnea, insomnia, decreased appetite, constipation, diarrhea and financial difficulties. A high score on each of these scales represents a high level of symptoms or problems in the patient. The QLQ-CR29 questionnaire was developed following revision of the QLQ-CR38, and has been demonstrated internationally to have both sufficient validity and reliability to support its use as a supplement to the EORTC QLQ-C30 for assessing patient-reported outcomes during treatment for colorectal cancer in clinical trials and other settings. The QLQ-CR29 questionnaire contains 29 items, 18 of which address gastrointestinal symptoms, pain and problems with micturition, and separate scales are available for participants with or without a stoma, while separate items address sexual function in men or women. The response categories for each item are the same as those used in the QLQ-C30. This questionnaire has also been validated in Spain [23, 24].

The HADS questionnaire is designed specifically for individuals with a physical illness. It is divided into two subscales, with seven questions pertaining to symptoms associated with anxiety and seven with depression. Each of the 14 items consists of a 4-point Likert scale (ranging from 0 to 3) that applies to the previous week. A total score for each subscale is then calculated, ranging from 0 to 21 [25]. The HADS has been translated into and validated in Spanish [26].

The Duke-UNC FSSQ questionnaire is composed of 11 items, each of which is rated on a five-point Likert scale ranging from 1 to 5: the higher the score, the better the perceived social support. It assesses subjective social support in two domains, Confidant support: 6 items (score range 6–30) and Affective support: 5 items (score range 5–25), and provides an overall social support measure (score range: 11–55) [27]. The questionnaire has been translated into and validated in Spanish [28].

The BI questionnaire consists of 10 items that assess the ability to perform certain basic activities of daily life without help. It includes 2- and 4-point response options that produce a score ranging from 0 (completely dependent) to 100 (completely independent), with intervals of 5 points [29]. The BI has been translated into and validated in Spanish [30].

Patients were first contacted upon inclusion in the surgical waiting list for a surgical intervention or were interviewed during their hospital admission period. Questionnaires were subsequently provided at 30 days, and at 1, 2, 3 and 5 years after surgery. In all instances, a reminder letter was sent to patients who had not replied after 15 days. Those who had not responded after a further 15 days received another reminder letter and, where feasible, contacted by telephone.

Questionnaires for the 30 day follow up visit were sent to patients attending the following hospitals: Hospital Costa del Sol, Hospital Valme, Hospital Virgen del Rocío, Hospital Virgen de las Nieves, Corporació Sanitaria Parc Taulí, Hospital Universitario La Paz, Hospital Infanta Sofía, Hospital Clínico San Carlos, Hospital Fundación Alcorcón, Hospital Basurto, Hospital Cruces, Hospital Donostia, Hospital Galdakao-Usansolo, Hospital Txagorritxu.

Figure 3 illustrates the data gathering process established for the entire follow-up period.

### Safety considerations

There is no explicit intervention in this study. Nevertheless, due to the sensitive nature of the disease and for reasons of confidentiality, all information provided to patients, either directly by the researchers or reviewers or included in the questionnaires, avoid references to cancer or tumors, referring instead, to “your bowel problem”. Patients may refuse to participate at any time during the study, whether during recruitment or follow-up. Furthermore, patients who prefer to complete the questionnaire by phone are permitted to do so. All participating centers have referring staff available for questions that the patient or patient’s family may have about the research project.

### Follow-up

Follow-up visits up to two years will be performed at all 22 participating hospitals. Due to financial restrictions and organizational resources, 3- and 5-year follow-up visits will be restricted to 18 hospitals (Hospital de Antequera, Hospital Costa del Sol, Hospital Universitario de Valme, Hospital Universitario Virgen del Rocío, Hospital Universitario Virgen de las Nieves, Hospital Universitario de Canarias, Corporació Sanitaria Parc Taulí, Hospital del Mar, Hospital Clínico San Carlos, Hospital Universitario La Paz, Hospital Infanta Sofía, Hospital Universitario Fundación Alcorcón, Hospital Galdakao-Usansolo, Hospital Universitario Araba, Hospital Universitario Basurto, Hospital Universitario Cruces, Hospital Hospitalario Donostia, and Hospital Universitario Doctor Peset).

### Data management and statistical analysis

A. Sample size selection

Studies of predictive model development indicate that is necessary to include at least 10 events of the dependent variable of interest (in this case: mortality, major complications, relapses or readmissions) for each independent variable included in the multivariate logistic regression model [31, 32]. Therefore, we estimated that at least 100 events of the dependent variable in the sample are required in order to ensure that the regression model would adequately converge. Previous data indicate that the number of events of the dependent variable mortality would be > 15 % of patients operated on in the first year, higher that the expected percentages of other parameters. We therefore estimated that more than 300 events of any of the dependent variables of interest should be included.

Sampling: we consecutively collected all new cases until the sample size was achieved.

B. Missing data assumptions and recoding of variables

#### Tumor location

Tumor location was categorized as follows: the appendix, caecum, ascending colon, hepatic flexure and transverse colon were defined as the right colon, whereas the splenic flexure, descending colon, sigmoid and rectosigmoid were defined as the left colon. Rectal tumors were also recorded.

#### Symptomatology

Patient pre-intervention disease-specific symptoms were collected. Where missing observations were observed for any recorded symptom, it was assumed to be asymptomatic. A new variable was created according to the presence/absence of these symptoms to determine whether the patient had experienced any type of symptomatology or not.

#### Complications

A list of several complications (yes/no answers) were collected throughout the course of the follow-up period (intra-surgical, during the hospital admission post-surgical, and at 30 days and 1, 2, 3 and 5 years after the intervention). If missing data were observed for each item in the aforementioned list, no complication was considered to have occurred. Subsequently, at each measurement point, an overall dichotomous variable was defined to classify patients as with and without complications. Complications were also classified into different types, infectious, hematological, surgical or medical.

#### Surgical severity

We redefined the surgical severity variable into three categories (moderate, major or complex major) depending on the type of procedure used to excise the tumor or to palliate symptoms.

The moderate severity category referred to transanal endoscopic microsurgery (TEM) or discharge colostomy; the major severity category to surgical techniques such as right/left hemicolectomy, any colectomy, total proctocolectomy, sigmoidectomy or transanal resection; and the complex major severity category corresponded to anterior resections or abdominoperineal amputation. Any intervention that involved a surgical colorectal technique, in addition to another procedure that affected any other organ, was considered a complex major surgical intervention.

We did not consider any colorectal cancer surgeries as belonging to the minor severity category.

#### pTNM staging

TNM classification was assessed according to the American Joint Committee on Cancer (AJCC), 7th Edition 2010 [14], and focused on the pre-intervention/clinical (cTNM) and the histopathological report (pTNM). For the final stage grouping, pTNM was considered. When only partial pTNM was available, a combination of cTNM and pTNM data were considered: (a) where the pM component was not recorded, pM was replaced by the corresponding cM stage or, when the latter was missing, pM was assumed to be pM0; (b) in subjects whose pN component was unobserved, the pTNM was classified as at least stage III if there was evidence that lymph nodes were infiltrated. Otherwise, pTNM was specified as stage II; and (c) tumors without pT value described pN0-pNx and pM0-pMx parameter values, where pTNM was therefore deemed to be stage II.

When pTNM was fully unobserved, it was replaced by the analogous cTNM data. If the latter information was also missing, it was recorded as a missing value.

C. Statistical Analysis

In relation to the different study objectives, exploratory analysis of the population sample will be performed using mean and standard deviations for continuous variables (or median and interquartilic ranges, when the observed variables do not follow the normality distribution) and frequencies and percentages for qualitative variables.

Predictive models will be developed to address objectives 1 and 2. The overall sample will be randomly divided into derivation and validation sets. On the one hand, for binary outcomes (such as death, tumor recurrence, major complications, or readmission), multivariate hierarchical logistic models will be used. Goodness-of-fit of models will be assessed by means of the Hosmer–Lemeshow test (*p*-values > 0.05 indicate good model calibration) and the area under the Receiver Operating Curve (ROC), AUC, will be used to evaluate the discrimination ability of the final performance models. An AUC value > 0.80 will indicate that the model discriminates well. Furthermore, multivariate Cox regression models will be used to answer to those objectives. On the other hand, in cases of continuous outcomes (variables related to deterioration of quality of life) as with objective 3, the use of multivariate hierarchical linear mixed models is proposed. The intraclass correlation coefficient (ICC) will be calculated to assess the correlation among observations within a cluster by dividing the patient variance by the total sample variance. A small coefficient would indicate that patients and participant centers are independent.

For study endpoints related to health services research, the following statistical approaches will be used: for two-level outcomes, the Student’s t-distribution or the non-parametric Wilcoxon test (for non-normal distributions) will be applied. Where there are three or more categories in the outcome, ANOVA analysis or a Kruskal–Wallis approach (for non-normal distribution) are the proposed tests. Otherwise, for categorical variables, the Chi-square test (or Fisher’s Exact method, where required) will be used. Multivariate models will be used as needed for appropriate adjustments. For some analyses, subgroups based on tumor location (colon or rectum) will be identified.

An analysis of comparative costs between two alternative surgical approaches (open and laparoscopic surgery) will be performed. For this purpose, generalized linear models (GLM) will be employed using the patient level cost as the dependent variable and taking clinical covariates into account (age, sex, stage, among others). The statistical model will determine which covariates are associated with higher costs, as well as any significant differences in cost between the two alternatives. Analysis will be conducted at 2 and 5 years of follow up.

Finally, results related to treatment will be a valuable source of information for inclusion in a discrete-event simulation model reproducing a colorectal cancer-screening program. Clinical and quality of life data for treatment of colorectal cancer detected by screening or clinical approaches will be useful to assess not only the impact of screening, but also to compare different treatment strategies through a cost-effectiveness or budget impact analysis.

### Quality assurance

Reviewers were instructed in the identification of pertinent data and a specific manual was developed to aid in data collection. All data is maintained in an “ad hoc” database. Personal patient data that may allow identification of patients will be maintained separately from the main database, where all clinical and PRO data are included. To access the database, specific usernames and passwords are required. A log has been developed to monitor database access. Databases are located in six places in the servers of participating hospitals. Each hospital has a designated person in charge of the supervision of the data collection who serves as a referral contact for the reviewers. A project manager coordinated the study in the initial 2 years, with study coordination checks performed every 6 months to evaluate the quality and completeness of the data and request additional information and corrections if needed. Twice yearly, the study investigators meet to review the status of data collection and any issues that arise.

### Duration of the project

The entire project is planned to last for more than 7 years (2 years for recruitment and 5 for follow-up until the end of 2017), until the data from the last patient has been collected and entered into the database. Following this, a 6-month period of error correction and final database preparation is anticipated. Manuscript writing may commence at intervals of 1, 2, 3 and 5 years of follow-up, once errors have been corrected and agreed on by the coordination team.

### Project management

Seven study leaders are responsible for each goal and in different geographical areas, along with one coordinator. Dr M. Bare was tasked with the development of prediction models to determine risk factors of adverse events in the short-term. Dr J.M. Quintana is responsible for objectives 2 (to determine risk factors of death, tumor recurrence, major complications, readmission or deterioration of quality of life in the mid-term) and 3 (evaluation of patient reported outcomes from before the intervention to the end of the follow-up period). He is also the coordinator of the entire study. Dr M. Redondo is responsible for objective 4 (to study the role of biological markers in the prediction of similar adverse results). The goals related to health services research are assigned to the following: C. Sarasqueta (equity), E. Briones (quality of care and appropriateness), A. Escobar (evaluation of psychosocial support interventions) and N. Fernandez-de-Larrea (cost of various surgical approaches). Dr M. Redondo and Urko Aguirre, acting as coordinator, are responsible for the follow-up period to 3 and 5 years. The leaders of each study goal, in conjunction with the coordinator, comprise the coordination committee responsible for all decisions.

### Ethics

All patients were informed of the objectives of the study and invited to voluntarily participate at the first contact and at the 3-year follow-up visit. Patients who agreed to participate provided written consent. Two types of informed consent forms were created, one for the entire sample and one for the subsampling cohort, where biological data was requested. All information was kept confidential. The Institutional Review Boards of the participating hospitals approved this study.

## Discussion

As illustrated in Fig. 2, by the time of manuscript preparation, 2749 patients had been recruited according to the study protocol described previously, 1980 of which had been diagnosed with colon cancer and 769 with rectal cancer. This population represents a relatively large cohort of colorectal cancer patients for prospective follow up. Description of the basic characteristics of the sample, stratified by colon or rectal cancer diagnosis, is displayed in Table 2.

### Anticipated issues

The main limitations of this type of study design are those related to missing patients as a result of an extensive follow-up period (5 years, in this instance) and, at the same time, missing data related to some variables, especially those related to patient questionnaires. To minimize these risks, all patients are informed of the objectives of the study prior to enrollment and again during the follow-up visits. Furthermore, to reduce missing data from questionnaires, up to three letters are sent to patients and conversion to phone visits is available upon request. Given the lengthy follow up period, regular patient contact are established. Furthermore, the contact physicians at each hospital remind patients of their participation in the study where possible.

An additional issue is the reliability of data gathering by reviewers. To standardize the collection of data and avoid errors, all reviewers are adequately trained and have received specific instructions on how to collect data for each variable.

### Expected outcomes of the study

The identification of potential risk factors for adverse events (death, major complications, relapse, reoperations or readmission) in the short- and medium-term has undeniable practical value from a clinical perspective. These data will assist clinicians to identify and predict possible unwanted outcomes, establish preventive measures, if feasible, to correct these outcomes should they occur, and provide patients with a medium-term prognosis. In addition, the development of simple clinical prediction rules that are easy-to-implement in daily practice on desktop computers, tablet, mobile phones, and other devices may facilitate clinicians in making decisions to prevent such occurrences.

The large cohort of patients who have undergone surgery for colon or rectal cancer followed by 5 years of post-surgical follow up may provide useful information to help address other health services research questions.

### Dissemination of results and publication policy

For the purpose of this study, a research group called REDISSEC CARESS-CCR (Results and Health Services Research in Colorectal Cancer)- has been established, and reflects the primary research participants of each center. For publication policy purposes, an author has to fulfill the necessary requirements by contributing to each of three activities: 1) conception/design and/or analysis/interpretation, 2) writing, and 3) approval of final version, and take public responsibility for the content of the paper. All co-authors have to review and agree with the contents of the manuscript as submitted. Due to the study objectives, the entire study and manuscripts will follow the STROBE guidelines for conducting and disseminate observational studies and the TRIPOD statement for reporting of a multivariable prediction model for individual prognosis or diagnosis [33, 34].

The main study results will be disseminated in the media, to clinicians, managers and policy makers in the proper format.

## Additional file

Additional file 1: Table S1. Grouping of complications. **Table S2.** Chemotherapy information at the beginning of the study. (DOCX 17 kb)
