# Supplementary material for: Colorectal cancer health services research study protocol: the CCR-CARESS observational prospective cohort project
Source: BMC Cancer. 2016 Jul 8;16:435. doi: 10.1186/s12885-016-2475-y (PMC4939051; doi:10.1186/s12885-016-2475-y)
Supplement: Additional file 1: Table S1. — Grouping of complications. Table S2. Chemotherapy information at the beginning of the study. (DOCX 17 kb) [file 12885_2016_2475_MOESM1_ESM.docx]

**Table S1.** Grouping of complications

|  |  |
| --- | --- |
| Total |  |
|  |  |
| Infectious |  |
| - Pneumonia |  |
| - Infection because of catheter |  |
| - Urinary tract infection |  |
| - Septic shock |  |
| - Localized intra-abdominal infection (abscess) |  |
| - Diffused intra-abdominal infection (peritonitis) |  |
| - Fever of unknown origin (FUO) |  |
| - Infection of surgical superficial location |  |
| - Infection of deep surgical location |  |
| - Respiratory tract infection |  |
| Hematological (Haemorrhagical /Thrombosis/Embolism) |  |
| - Haemorrhage of the injury |  |
| - Upper gastrointestinal (GI) bleeding |  |
| - Internal bleeding of other organs |  |
| - Hemoperitoneum |  |
| - Ictus |  |
| - Deep vein thrombosis (DVT) |  |
| - Pulmonary thromboembolism (PTE) |  |
|  |  |
| Surgical |  |
| - Seroma |  |
| - Dehiscence of suture |  |
| - Anastomotic dehiscence |  |
| - Evisceration |  |
| - Urine retention |  |
| - Necrosis (abdominal wall) |  |
| - Enterocutaneous fistula |  |
| - Biliary peritonitis |  |
| Medical |  |
| - Heart failure  - Angina or acute myocardial infarction |  |
| - Kidney failure |  |
| - Respiratory insufficiency |  |
| - Liver failure |  |
| - Neurological alterations (except ICTUS) |  |
| - Hypotension |  |
| - Hypocalcemia |  |
| - Anaemia |  |
| - Intestinal obstruction |  |
| - Paralytic ileus |  |
|  |  |

**Table S2**. Chemotherapy information at the beginning of the study

|  |  |
| --- | --- |
| Combinations of drugs |  |
| - Folfox only |  |
| - Folfiri only |  |
| - Capox/Xelox only |  |
| - Fluorouracil only (FL) |  |
| - Fluorouracil - Leucovorin only (FL-LV) |  |
| - Monotherapy only |  |
| - Folfox + Folfiri |  |
| - Folfox + Capox/Xelox |  |
| - Folfox + FL |  |
| - Folfox + FL-LV |  |
| - Folfox + monotherapy |  |
| - Folfiri + Capox/Xelox |  |
| - Folfiri + FL-LV |  |
| - Capox/Xelox + FL-LV |  |
| - Capox/Xelox + monotherapy |  |
| - Folfox + Folfiri + monotherapy |  |
| - Folfox + Capox/Xelox + FL-LV |  |
| - Folfox + Capox/Xelox + monotherapy |  |
| - Folfox + FL + monotherapy |  |
| - Folfox + Folfiri + Capox/Xelox + FL-LV |  |
